# Health Behaviors of Student Community Research Partners When Designing and Implementing a Healthy Lifestyle Intervention on College Campuses

**DOI:** 10.3390/bs8110099

**Published:** 2018-10-26

**Authors:** Makenzie L. Barr, Sarah E. Colby, Kristin Riggsbee, Krista Leischner, Anne E. Mathews, Melissa J. Vilaro, Kendra K. Kattelmann, Melissa D. Olfert

**Affiliations:** 1 Division of Animal and Nutritional Sciences, School of Agriculture, Davis College of Agriculture, Natural Resources and Design, West Virginia University, 1194 Evansdale Drive, G25 Agriculture Sciences Building, Morgantown, WV 26506, USA; mbarr6@mix.wvu.edu; 2 Department of Nutrition, The University of Tennessee, 1215 W. Cumberland Avenue, 229 Jessie Harris Building, Knoxville, TN 37996-1920, USA; scolby1@utk.edu (S.E.C.); kristin.riggsbee@gmail.com (K.R.); 3 Department of Health and Nutritional Sciences, South Dakota State University, HNS Department, Rotunda Lane, Wagner 425, Box 2203, Brookings, SD 57007, USA; krista.leischner@jacks.sdstate.edu (K.L.); kendra.kattelmann@sdstate.edu (K.K.K.); 4 Food Science and Human Nutrition Department, University of Florida, 572 Newell Dr., 359 FSHN Building, P.O. Box 110370, Gainesville, FL 32611-0370, USA; anne.mathews@ufl.edu (A.E.M.); mgraveley@ufl.edu (M.J.V.)

**Keywords:** CBPR, college students, health, behavior, interventionists

## Abstract

Few studies work with college students as equal partners in all aspects of Community-Based Participatory Research (CBPR) and even less evaluate behaviors of those college partners. The current study aimed to examine health behaviors of students by designing and implementing a peer-led, social marketing campaign (Get Fruved) to promote healthier lifestyles on their campuses. Enrolled students (n = 376) were trained to either design and implement a health promotion intervention (Social Marketing and Environmental Interventionists; SMEI, n = 78), be peer mentors (PM; n = 205), or serve as control participants (n = 93). Students’ behaviors (dietary, activity, and stress) and anthropometrics were assessed at baseline, 6 months, and 12 months. The population was predominately Caucasian, female, and between 19 and 20 years old. On average, fruit and vegetable consumption slightly decreased across all time points for each group with control at a larger decline. Students International Physical Activity Questionnaire (IPAQ) scores showed students met recommended amounts of activity throughout the intervention, with males reporting higher activity levels. Cohen’s Perceived Stress Scale (PSS) analyses indicated 19 year olds had higher stress along with females had higher than males. Students involved in a CBPR approach to be trained, design, and implement a lifestyle intervention can achieve maintenance of health behaviors throughout a college year when compared to control students.

## 1. Background

Community-Based Participatory Research (CBPR) brings together the efforts of researchers and community members as equal partners in developing an intervention for that community [1,2]. An important aspect of CBPR is working with partners who are invested in the research and want to make a difference in their community. One form of CBPR is for researchers to team with college students to conduct an intervention on a college campus. Various research programs have used college students either to gain insight into a collegiate population, develop and/or implement an intervention, or to assist with data collection [2,3,4,5,6,7,8,9,10,11,12,13,14,15,16,17,18]. However, few of these studies have worked with students as equal partners in all aspects of a lifestyle intervention research process, as is called for in CBPR, and fewer still have evaluated the health impacts made on the interventionists. Previous literature has only involved student partners in aspects of intervention development such as focus group only, data collection only, or data analyses only. Our project examined the use of CBPR student partners in training on the subject, development of the intervention, marketing and events, as well as dissemination of the intervention.

One field that offers significant potential for a successful CBPR college campus intervention is in the areas of health and wellness. College students live in an independent environment on campus which has been noted to be tied with poor dietary, activity, sleep, and stress behaviors and is a target for health promotion interventions [19,20,21,22,23]. However, identifying students interested in health-related fields could serve as successful collaborators on leading CBPR healthy lifestyle interventions for their peers [2,4,8,15,16,24]. Currently, there is little research investigating behavior change in these community interventionists who design and implement a CBPR intervention. Furthermore, participating in the process of designing and conducting a healthy lifestyle intervention could improve the interventionists’ health-related behaviors and attitudes. However, to date, no study has investigated the changes among college student research partners in a CBPR healthy lifestyle intervention compared to control students who were not engaged in the intervention’s design and implementation.

## 2. Project Overview

Students described in this manuscript were partners in the development of the ‘Get Fruved’ study (short for FRUits and VEgtables). Get Fruved was a multi-state, peer-led, social marketing, and environmental change obesity prevention intervention funded by the United States Department of Agriculture. The project involved four control (Syracuse University, Kansas State University, University of Maine, and Auburn University) and four intervention universities (University of Tennessee, South Dakota State University, University of Florida, and West Virginia University) in the United States. Get Fruved used CBPR to increase healthy lifestyles among the college population, specifically, first-year students at higher risk for weight gain and other unhealthy lifestyle behaviors [25].

Students interested in healthy living at each of the four intervention university campuses were recruited using various methods [26]. A recruitment goal of 30 Social Marketing and Environmental Intervention students (SMEI students) and 120 Peer Mentors for Health (PM) per intervention university was set. SMEI and PM were recruited throughout fall semester of 2014 to enroll in a university course (SMEI: 3 credit hours; PM: 1 credit hour) during spring 2015. Recruitment was performed through campus-wide announcements, social media, flyers, word of mouth, informational meetings, and campus informational booths. In addition, recruiters emailed student organizations, spoke to 100/200 level courses, designed short recruitment videos, and reached out to the universities’ health organizations. Upperclassmen students (students enrolled as sophomore-senior status or had 1–3 years of experience on campus) were preferentially recruited because they were familiar with the territory, were on campus the upcoming year to help implement the intervention, and understood the need for a lifestyle intervention at their university. These upperclassmen CBPR partner students had the ability to decide what program (SMEI or PM) they were interested in taking.

During the 15-week course, SMEI students were trained to develop a peer-led social marketing campaign intervention focused on three main topic areas: diet, physical activity, and stress management [27]. Within Get Fruved, these areas were targeted as specific zones of healthy living that can impact well-being long term. An important aspect of the training program included SMEI students auditing the healthfulness of their environment. From this audit and awareness of their campus, teams were formed to design a week of intervention events for the Fruved intervention toolkit. During this semester, PM students were simultaneously trained to mentor incoming first year students on the three main umbrella topic areas as well as aspects of the campus including residence life and financial help, leadership, communication, counseling and behavior change, crisis management, and conflict resolution. In this Fruved intervention feasibility pilot year, PMs would be matched to incoming first-year Fruved students and encourage them to make healthy choices, and attend the SMEI students Fruved events, in the frequency and mode (texting, calling, meetings, email, or other) by which their mentee student chose. Two of the control universities (Syracuse University and University of Maine) recruited students to serve as controls. The multi-state umbrella Institutional Review Board at University of Tennessee, Knoxville, approved the study protocol for University of Tennessee, West Virginia University, and South Dakota State University (IRB approval #14-09366 B-XP). The University of Florida IRB approved the same strategies for activities at the University of Florida (IRB approval #2014-U-0547FRUVED). The IRB approved study procedures for the control universities, Syracuse University and the University of Maine (#14-175 and #2014-06-21, respectively). This study was prospectively registered on clinicaltrials.gov, NCT02941497.

## 3. Purpose

The purpose of this article is to describe changes in self-reported behaviors of SMEI and PM after training, designing, and implementing the Get Fruved pilot intervention [27] compared to control students. To the best of our knowledge, this is the first study assessing the behavior change of young adult community partners across the course of designing and implementing a health promotion intervention for their peers.

## 4. Methods

### 4.1. Participants

In fall of 2014, intervention and control students from 8 universities were recruited to be enrolled in the Get Fruved pilot project [26]. Inclusion criteria required participants were at least 18 years of age and upperclassmen with familiarity of their campus and understanding of the needs of the campus community. These criteria were in place to ensure that these students could inform and design an appropriate intervention that is feasible on their campus by having been on campus for at least one year. Control students were recruited through professors, listservs, flyers, and announcements on campus to serve as a Get Fruved control participant and provide data with the understanding of being compensated monetarily for their time. Control students did not complete the training course. All student participants completed health behavior surveys and anthropometric assessments prior to designing the intervention in spring 2015 (baseline), after designing the intervention in fall 2015 (6-months), and after implementation of the first year of the Get Fruved intervention in spring 2016 (12-months). All control and intervention students were invited to return for a follow-up assessment.

### 4.2. Outcome Measures

Trained researchers performed anthropometric measures at each assessment. Height was measured by stadiometer (Heightonic digital stadiometer; Issaquah, WA, USA) with the participant standing, facing forward without shoes, including height. Weight was taken without shoes and with light clothing via digital scale (Electronic Tanita scale; Arlington Heights, IL, USA). Body Mass Index (BMI) was calculated as weight in kilograms divided by height in meters squared. Waist, hip, and neck circumferences were taken while the participant was minimally clothed via Gulick meter (North Coast Medical Gulick tape measure; Gilroy, CA, USA). Waist measurement was taken at the midpoint between the lower margin of the last palpable rib and the top of the iliac crest; hip circumference was taken at the largest area of the hips, and neck circumference was taken at the Adam’s apple area of the neck, indicated by a swallowing motion of the participant. Blood pressures were taken in a seated resting position with an automated cuff (Omron HEM 907 XL Intellisense Prof. Digital BP monitor; Kyoto, Japan) that measured twice and averaged [28,29]. All measures were taken twice and averaged. Repeated measures were taken a third time if initial measures had a range larger than 0.2 kg for weight, 0.2 cm for height, and 1.0 cm for waist, hip, and neck circumference. Values were averaged for analysis.

Student participants completed self-reported behavioral surveys, including the National Cancer Institute’s Fruit and Vegetable Screener (NCI FV) [30], International Physical Activity Questionnaire (IPAQ) [31], and Cohen’s Perceived Stress Scale (PSS) [32]. As the three main foci of the intervention were diet, physical activity, and stress management, these tools were used as outcome measures. The validated NCI FV screener tool identifies 19 self-reported questions on daily fruit and vegetable intake (servings/day). Questions asked students’ type and amount of fruit and vegetable consumption during the past month (i.e., “Each time you ate lettuce salad, how much did you usually eat?”). The short-form IPAQ is a validated questionnaire to score physical activity metabolic equivalency tasks in minutes/week (METs). The tool is comprised of six self-reported items of type (light, moderate or vigorous), frequency in days, and duration in minutes of physical activity preformed (i.e., “During the last 7 days, on how many days did you do vigorous physical activities like heavy lifting, digging, aerobics, or fast bicycling?”, “How much time did you usually spend doing vigorous physical activities on one of those days?”). The validated PSS scores perceived stress with 14 self-reported questions on a scale of 0–56. Items include questions regarding stress in the past month (i.e., “in the last month, how often have you felt nervous or stressed?”).

### 4.3. Statistical Methods

All analyses were conducted using SAS (SAS^®^, Version 9.3) [33] and JMP (JMP^®^, Version Pro 11) [34]. An a priori sample size analysis to detect a medium effect size (0.25), 0.05 alpha value among 3 groups with 5 measures and a power of 80%, total sample size was 96 participants which was captured within this cohort. Demographic data are reported in frequencies and percentages and group differences at baseline (SMEI, PM, and control) were analyzed with chi-square or Fisher’s exact tests with alpha value set to 0.05. Outliers removed prior to analysis (n = 15). Descriptive statistics were run on baseline anthropometric measures; group differences of behavioral scores were tested via analysis of variance (ANOVA) for initial analysis. Finally, descriptive statistics were run on all outcomes at baseline and at the 6-month and 12-month follow-ups. All outcome distributions were examined and transformed as necessary, which included cubed root transformation to NCI FV and IPAQ outcomes in order to improve normality; group differences on the transformed data were tested via ANOVA analysis.

A series of mixed regression models were used to assess the cubed root transformed NCI FV and IPAQ outcomes, and the PSS behaviors in group, over time, and group by time interaction. To fit the flexibility of the means, individual variances and covariances of the data, as well as both fixed and random effects within each model, mixed linear models were used [35,36]. Covariate inclusion was decided a priori by selecting significant bivariate demographics for any of the outcomes and was consistently entered into all models. The best fitting model was determined using Akaike Information Criterion (AIC) and other goodness of fit indices. Null Model Likelihood Ratio chi-squared test (LRT) reported for model fit of covariance structure. Time was treated as a continuous variable in all models. The best fitting models for each PSS and FV included only a repeated statement of time, using an autoregressive (AR1) covariance structure, correcting for the degrees of freedom using Kenward–Roger method, and REML estimation, with student ID as the subject repeated variable. Best fitting model for IPAQ also included only a repeated statement of time, using an unstructured covariance structure, again correcting for the degrees of freedom using Kenward–Roger method, and REML estimation, with student ID as the subject repeated variable. For all models, pairwise comparisons with post hoc Tukey adjustment were performed for significant adjusted mean differences by group. Missing data was assumed to missing at random and pairwise deletion was utilized.

## 5. Results

The population was predominately Caucasian (*p* = 0.002), female (*p* < 0.0001), and between 19 and 20 years of age (*p* = 0.0007). Table 1 outlines the demographic differences between SMEI, PM, and Control groups. At baseline, weight, BMI, and hip, waist, and neck circumference were significantly different between PM and Control (all *p*’s < 0.01) while waist and neck circumference were significantly different between SMEI and control (*p* < 0.001) (Table 1). At 12-month assessments (data not shown), significance only remained between control and PM waist circumference (*p* = 0.0096) and neck circumference (*p* = 0.0004). Further, although no differences were seen at baseline, significant differences were seen at 12-months between control and SMEI systolic (*p* = 0.011) and diastolic (*p* = 0.026) blood pressures and control and PM systolic (*p* = 0.002) and diastolic (*p* = 0.017) blood pressures.

## 6. Behavior

### 6.1. Fruit and Vegetable Intake

Average baseline fruit and vegetable intake for SMEI, PM, and control was 3.7 ± 2.9, 3.9 ± 2.9 and 3.3 ± 2.5 servings/day, respectively (Table 2). From baseline to 12-month follow-up, fruit and vegetable consumption decreased across all groups. However, one-way ANOVA identify fruit and vegetable consumption in SMEI (−0.14 servings) and PM (−0.71 servings) remained stable across time while control had a significant decrease (−1.1 servings) by 12-months. After transformation of the variable to improve normality, significance was found from baseline to 12-months in SMEI (*p* = 0.0210) and control (*p* = 0.0012) but not within PM (*p* = 0.3023). One-way ANOVA results (Table 2) show significant differences among NCI FV servings between groups (*p* < 0.0001).

NCI FV null model LRT was significant (χ^2^ (1) = 19.47, *p* < 0.0001). Significant type 3 tests of fixed effects for race/ethnicity: F (3, 453) = 2.88, *p* = 0.0358. and time: F (1629) = 13.05, *p* = 0.0003, but not with gender (*p* = 0.26), age (*p* = 0.60), or group (*p* = 0.19) (data tables not shown for model type 3 fixed effects). Fixed effects significance was indicated within time (*p* = 0.0002) and ‘other’ race/ethnicity (*p* = 0.0108) (Table 3). No group by time interaction was detected (*p* = 0.26). After Tukey adjustment for multiple comparisons, a significant difference was seen only between PM (adj. mean = 2.5 servings) and control groups (adj. mean = 2.3 servings/day) (*p* = 0.0064).

### 6.2. Physical Activity

Baseline IPAQ scores for SMEI, PM, and control were 2876 METs, 2907 METs, and 3058 METs, respectively (Table 2). After a large increase in SMEI IPAQ scores at 6-month assessments, all groups IPAQ scores remained relatively the same from baseline to 12-month assessment. One-way ANOVA results (Table 2) significant differences among IPAQ METs between groups (*p* = 0.0055).

IPAQ null model LRT was significant (χ^2^ (5) = 30.79, *p* < 0.0001). Significant type 3 tests of fixed effects for gender: F (1, 444) = 12.02, *p* = 0.0006, and group: F (2503) = 3.95, *p* = 0.0199, but not with age (*p* = 0.33), race/ethnicity (*p* = 0.66), or time (*p* = 0.81). No group by time interaction was detected (*p* = 0.92). Table 3 indicates significance between gender (*p* = 0.0002) and SMEI (*p* = 0.0093). After Tukey adjustment for multiple comparisons, SMEI (adj. mean = 14.8) also had significantly higher IPAQ score than control (adj. mean = 13.7) (*p* = 0.0013). Further, males (adj. mean = 15.38 METs) had higher IPAQ scores than females (adj. mean = 14.15 METs) (*p* = 0.0002).

### 6.3. Perceived Stress

Baseline averages of PSS for SMEI, PM, and control were 23.6, 22.7, and 27.6, respectively (Table 2). Control participants PSS was higher at baseline and remained higher across all time points. One-way ANOVA results (Table 2) significant differences among PSS between groups (*p* < 0.0001).

PSS null model LRT was significant (χ^2^ (1) = 27.66, *p* < 0.0001). Significant type 3 tests of fixed effects for gender: F (1, 482) = 6.09, *p* = 0.0139, age: F (3614) = 6.62, *p* = 0.0002, group: F (2, 576) = 16.92, *p* ≤ 0.0001, time: F (1584) = 5.79, *p* = 0.0164, and approaching significance for race/ethnicity: F (3, 457) = 2.51, *p* = 0.0581. No group by time interaction was detected (*p* = 0.25). After Tukey comparison test, PM (mean = 22.93) had a better PSS than control (mean = 27.04) (*p* = 0.0001) and SMEI (mean = 23.43) had a better PSS than control (*p* = 0.0192). Further, males (mean = 23.04) had a better PSS than females (mean = 24.22) (*p* = 0.0177).

## 7. Discussion

The aim of the present study was to investigate health behavior changes among college student research partners in a CBPR healthy lifestyle intervention compared to control students who were not engaged in the intervention’s design and implementation. The intervention groups (SMEI and PM) started with and maintained healthier lifestyle habits compared with the control group throughout the year-long study. On average, while all groups showed reductions in fruit and vegetable consumption across the 12 months, the control group demonstrated the largest decrease. Similarly, the intervention groups reported higher activity and lower stress scores than the control group.

The American College Health Association-National College Health Association (ACHA-NCHA) 2016 survey reports the majority of undergraduate students in the United States are only consuming 1–2 servings of fruits and vegetables per day (61% were getting 1–2 servings per day, 27% were getting 3–4 serving per day) [37]. Although the student partners enrolled in the Get Fruved study did not meet recommended daily intake of fruits and vegetables (2015–2020 Dietary Guidelines for Americans and the United States Department of Agricultures’ MyPlate recommends five servings of fruit and vegetables per day for a healthy balanced diet [38,39]), their average intake was higher than the national average at completion of the intervention in both SMEI (3.6 servings) and PM (3.1 servings) groups. More specifically, in fruit and vegetable cup servings per day, we identified a decline in servings across the year in PM (baseline 3.9 cup servings per day; after 12 months 3.1 cup servings per day) and SMEI (baseline 3.7 cup servings per day; after 12 months 3.6 cup servings per day) students that was less than that of their control (baseline 3.3 cup servings per day; after 12 months 2.2 cup servings per day) counterparts. Further analysis via LMM analysis identified, after Tukey adjustment, PM had significantly higher FV servings compared to control, in relation to time.

Decreased physical activity has been linked to numerous medical conditions, including cardiometabolic disease such as cardiovascular disease and diabetes [40]. Despite this link, only 50% of USA adults meet physical activity recommendations of 150 min per week of moderate-vigorous activity [41,42,43] and college students, in particular, engage in aerobic exercise less than 3 times per week [44]. In terms of IPAQs MET minutes per week, the recommended 150 min of moderate to vigorous physical activity per week is equal to the range of 500–1000 METs (depending on intensity) [43]. In the present study, participants averaged over 2000 MET minutes per week at all time points, indicating they met physical activity recommendations throughout the duration of the study. LMM analysis showed both PM and SMEI had greater activity levels than control in relation to time. Further, males had higher activity than females which his supported by previous findings [45,46].

In contrast to Bieter, et al. who identified transfer students, upperclassmen, and those living off-campus to be the students with the highest self-reported stress levels [47], the present study found students 19 years of age to have higher stress levels than the older students. Using Cohen’s PSS, Civitci determined the mean stress score of an average college student was 30.4 [48]. In the present study, both intervention groups, as well as the control group, reported mean stress scores from 21.2–27.6 across all time points. However, we do acknowledge that average PSS was higher at 12 months than baseline but could be attributed to 12-month assessments being taken near university final exams. Further LMM analysis showed both PM and SMEI had significantly lower stress scores than control, males lower than females, and Non-Hispanic White participants lower than Non-Hispanic Black and Other (including biracial). These results suggest students in the present study may have been better at managing their stress than the average college student due to a selection bias of individuals who volunteered to participate. Previous literature has shown that racial minorities experience higher stress and females feel more academic pressure, which increases stress, despite reporting higher levels of support from family and friends compared to males [49,50,51]. However, although our population was largely female, ethnicity was predominately Caucasian, as well as having students enrolled that are interested in health and designing a health intervention for their peers. 

When examining specific studies who observe CBPR based student partners, limited information is available on health behaviors among partners who are developing and engaging in intervention dissemination.

Some limitations are found within this study. First, we did find some drop-out among groups at each time point. This may be due to time, location on campus, or subsequent drop out from college. Second, our cohort was relatively healthy at baseline: they met physical activity recommendations and had higher fruit and vegetable consumption and lower stress levels than average college students. Therefore, they were less likely to make significant behavior improvements across this one-year period. This limitation is attributed to selection bias as students interested in participating in a CBPR healthy lifestyle research study were likely to be practice healthy lifestyle choices at baseline. Likewise, though, control students were recruited to be a control student in a health intervention, give anthropometric and behavior data, and knew the program. This allowed for a more comparable sample of cohorts between intervention and control groups. Had student participants been more reflective of a general college population, efforts to improve healthy lifestyle behaviors may have elicited more substantial changes. However, the aim of the overall study was to successfully implement a healthy lifestyle CBPR which students enrolled would be interested in healthy living. We recognize this as a limitation because findings are not generalizable to the average college population, specifically the large population of students who are not interested and do not prioritize healthy lifestyle behaviors and the promotion of such lifestyles. Furthermore, control students began a baseline with a higher BMI and waist circumference. This placed them at a disadvantage to those in the intervention groups and future studies warrant intervention and control anthropometrically matched groups for comparison.

Several studies have used college students to gain insight into a collegiate population, develop and/or implement an intervention, or to assist with data collection [2,3,4,5,7,8,9,10,12,13,14,15,16,17,18,24] but to the best of our knowledge, ours is the first to examine changes among college student research partners in a CBPR healthy lifestyle intervention compared to control students who were not engaged in the intervention’s design and implementation.

## 8. Conclusions

In this reasonably healthy group of students, health behaviors remained relatively stable across group and time during the 12 months, specifically in intervention groups. Specifically, we found fruit and vegetable consumption slightly declined, along with moderately higher perceived stress and stable physical activity. With these moderate changes across this year pilot intervention, it leads us to believe that being part of the design and implementation of a peer-led, health promotion intervention helped these students sustain their healthy behaviors over the year and attenuated a decline seen within control subjects and among typical college student populations.

## Figures and Tables

**Table 1:** Baseline demographics and anthropometrics of Social Marketing and Environmental Interventionists (SMEI), peer mentors (PM), and control. GPA: Grade point average; BMI: Body Mass Index.

Variable		n	SMEI	n	PM	n	Control	*p*-Value
*Demographics*								
Sex	Male	75	12 (16)	194	22 (11.3)	92	30 (32.6)	<0.0001 *
	Female		63 (84)		172 (88.7)		62 (67.4)	
Age	18	72	6 (8.3)	184	21 (11.4)	85	7 (8.2)	0.28
	19		26 (36.1)		50 (27.2)		32 (37.6)	
	20		29 (40.3)		63 (34.2)		28 (32.9)	
	≥21		11 (15.3)		50 (27.2)		18 (21.2)	
Race	Caucasian only	73	48 (65.8)	182	138 (75.8)	85	46 (54.1)	0.0022 *^,†^
	African American only		3 (4.1)		8 (4.4)		12 (14.1)	
	Hispanic/Latino only		22 (30.1)		36 (19.8)		27 (31.8)	
	Other including bi-racial		20 (24.1)		36 (43.4)		27 (32.5)	
Year	Freshman	74	8 (10.8)	183	41 (22.2)	92	17 (18.5)	<0.0001 *
	Sophomore		48 (64.9)		56 (30.3)		48 (52.2)	
	Junior		13 (17.6)		75 (40.5)		6 (6.5)	
	≥Senior		5 (6.8)		13 (7.0)		21 (22.8)	
GPA	3.5–4.0	70	25 (35.7)	184	91 (49.5)	90	36 (40.0)	0.042
	3.0–3.49		35 (47.3)		71 (38.6)		33 (36.7)	
	≤2.9		10 (14.3)		22 (12.0)		21 (23.3)	
*Anthropometrics*								
	Height (cm)	72	166.4 (8.0)	194	166.0 (7.6)	92	167.2 (9.6)	0.4991
	Weight (kg)	72	65.8 (13.4)	193	63.0 (11.8) ^†^	92	68.7 (16.5) ^†^	0.0040 *
	BMI (kg/m^2^)	72	23.6 (3.7)	193	22.8 (3.6) ^†^	92	24.4 (4.3) ^†^	0.0060 *
	Waist Circumference (cm)	72	76.1 (9.1) ^‡^	194	74.1 (8.2) ^†^	92	80.3 (13.5) ^†,‡^	<0.0001 *
	Hip Circumference (cm)	72	99.4 (8.5)	194	97.2 (7.9) ^†^	92	100.8 (10.2) ^†^	0.0035 *
	Neck Circumference (cm)	72	32.7 (2.9) ^‡^	194	32.0 (2.5) ^†^	92	34.2 (3.6) ^†,‡^	<0.0001 *
	Systolic Blood Pressure (mmHg)	72	112.5 (13.8)	194	110.8 (11.9)	92	109.0 (11.2)	0.1951
	Diastolic Blood Pressure (mmHg)	72	70.5 (8.7)	194	68.8 (7.8)	92	68.8 (8.2)	0.2912

Demographic data shown in frequency and percent; N (%). Anthropometric data shown in means (standard deviation); * *p* < 0.05, Chi Square analysis; ^†^ *p* < 0.05, Fisher’s Exact test when cell sizes were <5; *p*-value, One-way analysis of variance (ANOVA) by group; * *p* < 0.05: ^†^ significant differences between control and PM; ^‡^ significant differences between control and SMEI.

**Table 2:** One-way ANOVA of behavioral changes across time and group.

Variable		n	Baseline	n	6-Months	n	12-Months	*p*-Value ^a^
NCI FV (cup servings)	SMEI	77	3.7 (2.9)	35	3.1 (2.1)	37	3.6 (2.9) ^†^	<0.0001 *
	PM	203	3.9 (2.9)	131	3.4 (2.3) ^‡^	100	3.1 (1.9) ^‡^	
	Control	92	3.3 (2.5)	37	2.4 (1.8) ^‡^	61	2.2 (1.6) ^†,‡^	
IPAQ (METs)	SMEI	74	2876.3 (1813.6)	34	3410.2 (1704.1) ^†^	36	2791.2 (1608.0)	0.0055 *
	PM	196	2906.6 (1982.0)	125	2808.1 (1809.2)	99	2735.7 (1665.0)	
	Control	90	2772.6 (2592.6)	37	2385.5 (2242.3) ^†^	60	2830.1 (2765.8)	
PSS (score 0–56)	SMEI	70	23.6 (8.3) ^†^	32	21.4 (6.6) ^†^	34	25.2 (4.9) ^†^	<0.0001 *
	PM	183	22.7 (6.8) ^‡^	124	21.2 (6.6) ^‡^	98	25.5 (5.7) ^‡^	
	Control	81	27.1 (6.8) ^†,‡^	36	26.1 (6.5) ^†,‡^	58	27.6 (5.3) ^†,‡^	

Self-reported behavioral measurements were collected from all individuals at baseline and post intervention via survey. Untransformed baseline, 6-month, and 12-month measures reported in means and standard deviations (mean (SD)). ^a^ Transformed variables were used for further analyses and *p*-values in table. One-way ANOVA was used to analyze the main effects of group (SMEI, PM, and C). * *p* < 0.05; ^†^ *p* < 0.05 between SMEI and Control; ^‡^ *p* < 0.05 between PM and Control. NCI FV: National Cancer Institute’s Fruit and Vegetable Screener; IPAQ: International Physical Activity Questionnaire; PSS: Perceived Stress Scale

**Table 3:** Mixed Regression Models for Behavior.

Variable	Category	Estimate	SE	*t*-Value	*p*-Value
*NCI FV* (*cups/day*)					
Intercept		2.52	0.1	26.38	<0.0001 *
Gender (referent: male)	Female	0.02	0.02	1.28	0.2026
Age (referent: 18)	19	−0.01	0.03	−0.46	0.6469
	20	−0.03	0.02	−1.10	0.273
	≥21	−0.01	0.03	−0.44	0.6575
Race (referent: Hispanic/Latino only):	Non-Hispanic White	−0.03	0.03	−0.86	0.3881
	Non-Hispanic Black	0.03	0.05	0.55	0.5848
	Other (including biracial)	−0.09	0.04	−2.56	0.0108 *
Group (referent: control):	SMEI	0.01	0.03	0.33	0.7446
	PM	0.05	0.02	2.52	0.0123 *
BMI		0	0	−0.45	0.6546
Time		−0.07	0.02	−3.79	0.0002 *
Time * Role (referent: control):	SMEI	−0.03	0.03	−1.09	0.2752
	PM	0.04	0.02	1.86	0.0631
*IPAQ* (*METs*)					
Intercept		14.03	0.94	14.86	<0.0001 *
Gender (referent: male)	Female	−0.70	0.19	−3.71	0.0002 *
Age (referent: 18)	19	−0.41	0.26	−1.60	0.1101
	20	−0.14	0.24	−0.60	0.5507
	≥21	0.11	0.26	0.42	0.6769
Race (referent: Hispanic/Latino only):	Non-Hispanic White	0.31	0.33	0.94	0.3485
	Non-Hispanic Black	−0.10	0.5	−0.20	0.8449
	Other (including biracial)	−0.14	0.36	−0.38	0.7044
Group (referent: control):	SMEI	0.64	0.25	2.61	0.0093 *
	PM	0.24	0.19	1.24	0.2174
BMI		0.03	0.04	0.85	0.3943
Time		−0.01	0.18	−0.05	0.9641
Time * Role (referent: control):	SMEI	0.06	0.26	0.24	0.8108
	PM	−0.02	0.21	−0.09	0.9269
*PSS* (*score 0–56*)					
Intercept		24.13	2	12.07	<0.0000 *
Gender (referent: male)	Female	0.95	0.4	2.38	0.0177 *
Age (referent: 18)	19	1.62	0.53	3.06	0.0023 *
	20	−0.82	0.49	−1.67	0.0945
	≥21	−1.48	0.53	−2.77	0.0059 *
Race (referent: Hispanic/Latino only):	Non-Hispanic White	−1.57	0.67	−2.34	0.0194 *
	Non-Hispanic Black	−1.07	1.05	−1.02	0.3068
	Other (including biracial)	−0.13	0.73	−0.17	0.8616
Group (referent: control):	SMEI	−1.23	0.52	−2.35	0.0192 *
	PM	−1.63	0.41	−3.93	0.0001 *
BMI		0.02	0.08	0.3	0.7631
Time		0.97	0.36	2.66	0.0080 *
Time * Role (referent: control):	SMEI	0.22	0.53	0.42	0.6758
	PM	0.51	0.42	1.22	0.2214

Linear Mixed Model adjusted for age, gender, and race/ethnicity were used to analyze the main effects of group (SMEI, PM, and C), time, and their interaction on each behavior tool. Significant effects were followed by multiple comparisons using Tukey adjustment. *p*-values for the main effects and interaction are indicated. * *p* < 0.05. METs: metabolic equivalency tasks in minutes/week.

## Availability of Data and Materials

The datasets used and analyzed during the current study are available from the corresponding author on reasonable request.

## Abbreviations

CBPR	Community-Based Participatory Research
SMEI	Social Marketing and Environmental Intervention
PM	Peer Mentors for Health
NCI FV	National Cancer Institute’s Fruit and Vegetable Screener
IPAQ	International Physical Activity Questionnaire
PSS	Cohen’s Perceived Stress Scale
METs	Metabolic Equivalency Tasks-minutes/week
AIC	Akaike Information Criterion
LRT	Likelihood Ratio chi-squared test
AR1	Autoregressive
ACHA-NCHA	American College Health Association-National College Health Association
